# Recent advances in understanding inhibitor of apoptosis proteins

**DOI:** 10.12688/f1000research.16439.1

**Published:** 2018-12-03

**Authors:** Najoua Lalaoui, David Lawrence Vaux

**Affiliations:** 1 Cell Signalling and Cell Death, The Walter and Eliza Hall Institute of Medical Research, Melbourne, Victoria, 3052, Australia; 2 Department of Medical Biology, The University of Melbourne, Melbourne, Victoria, 3050, Australia

**Keywords:** IAP, cell death, innate receptors signalling, inflammation, smac-mimetic

## Abstract

The inhibitor of apoptosis proteins (IAPs) are a family of proteins that were chiefly known for their ability to inhibit apoptosis by blocking caspase activation or activity. Recent research has shown that cellular IAP1 (cIAP1), cIAP2, and X-linked IAP (XIAP) also regulate signaling by receptors of the innate immune system by ubiquitylating their substrates. These IAPs thereby act at the intersection of pathways leading to cell death and inflammation. Mutation of IAP genes can impair tissue homeostasis and is linked to several human diseases. Small-molecule IAP antagonists have been developed to treat certain malignant, infectious, and inflammatory diseases. Here, we will discuss recent advances in our understanding of the functions of cIAP1, cIAP2, and XIAP; the consequences of their mutation or dysregulation; and the therapeutic potential of IAP antagonist drugs.

## Introduction

The inhibitor of apoptosis proteins (IAPs) are a family of proteins that were first identified in insect baculoviruses ^1, 2^. These viral IAPs were found to block defensive apoptosis in order to facilitate viral replication ^1, 2^. Subsequently, cellular homologs have been identified in both invertebrates and vertebrates. Like viral IAPs, some cellular IAPs can inhibit apoptosis. Cellular and viral IAPs are characterized by the presence of baculoviral repeat domain (BIR) repeats. This review will focus on the most intensively studied mammalian IAPs, which are cellular IAP1 (cIAP1), cIAP2, and X-linked IAP (XIAP).

Mammalian IAPs were initially thought to inhibit cell death only by directly binding to caspases. However, only XIAP is able to bind caspase-3 and -9 ^3, 4^. Upon apoptotic stimuli, IAP inhibitors, including Smac/Diablo and HtrA2/Omi, are released from the mitochondria and bind to XIAP’s BIR domains, releasing active caspases into the cytosol ^5^. Unlike XIAP, cIAP1 and 2 are poor direct caspase inhibitors ^6^. Instead, they bind to tumor necrosis factor (TNF) receptor-associated factors (TRAFs) via their BIR1 domains ^7^ to block cell death induced by TNF receptor 1 (TNFR1) by promoting the activation of signaling pathways that induce the expression of pro-survival proteins.

Recent advances in understanding of IAP function from genetics, biochemistry, structural biology, and medicinal chemistry have shown that IAPs have roles beyond inhibiting cell death. All three IAPs have a carboxy-terminal RING (really interesting new gene) domain that allows them to act as ubiquitin E3 ligases that can ubiquitylate associated proteins as well as themselves. IAPs can regulate innate immune responses by limiting non-canonical nuclear factor kappa B (NFκB) signaling, promoting canonical NFκB and mitogen-activated protein kinase (MAPK) signaling, and inhibiting both caspase-dependent and -independent cell death. Drugs that antagonize IAPs, termed “Smac-mimetics”, have been developed to promote the death of cancer cells and those bearing intracellular infections. Use of these drugs in pre-clinical models has revealed additional roles of IAPs that might be exploited to treat certain inflammatory conditions and to enhance anti-tumor immunity.

## IAP and TNF signaling

### TNF-induced survival

**Figure 1.**
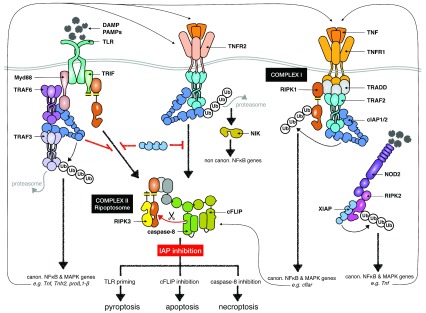
Regulation of innate receptor signaling pathways by inhibitor of apoptosis proteins (IAPs). Tumor necrosis factor (TNF) binding to TNF receptor 1 (TNFR1) triggers complex I formation, in which cIAP1 and 2 ubiquitylate RIPK1. This leads to the induction of canonical (canon.) nuclear factor kappa B (NFκB)- and mitogen-activated protein kinase (MAPK)-dependent genes, including *cFlar* encoding cFLIP. Subsequently, cytosolic complex II containing FADD, caspase-8, RIPK1, RIPK3, and cFLIP is formed. In this complex, cFLIP inhibits caspase-8 activation to block apoptosis and necroptosis. Inhibition of cIAP1 and 2 by Smac-mimetic drugs impairs canonical NFκB activation and accelerates the formation of complex II, which leads to apoptosis. When caspase-8 activation is blocked within complex II, RIPK1 and 3 are not cleaved and necroptosis is activated. Stimulation of nucleotide-binding oligomerization domain 1/2 (NOD1/2) receptors induces RIPK2 ubiquitylation by XIAP and activates the transcription of NFκB- and MAPK-dependent cytokines such as TNF, which amplifies the inflammatory signal. Binding of pathogen-associated molecular patterns (PAMPs) or damage-associated molecular patterns (DAMPs) to Toll-like receptors (TLRs) leads to the recruitment of the Myd88/TRAF3/6/cIAP1/2 complex. Within this complex, cIAP1 and 2 ubiquitylate TRAF3, inducing its degradation and increasing the expression of cytokines and chemokines. The other TLR adaptor, TRIF, recruits RIPK1 via its RIP homotypic interaction motif (RHIM) domain (yellow). Upon TLR activation, inhibition of IAPs by Smac-mimetics promotes the formation of the ripoptosome, which has a composition similar to that of complex II. TLR-induced expression of TNF and TNFR2 triggers cIAP1/2 degradation and a subsequent accumulation of NFκB-inducing kinase (NIK), which activates non-canonical (non canon.) NFκB-dependent genes. In the context of XIAP deficiency, the degradation of cIAP1 and 2 by TNFR2 leads to the formation of complex II. Activation of complex II or the ripoptosome can activate pyroptosis after TLR priming. TRAF, tumor necrosis factor receptor-associated factor.

Despite its name, TNF does not induce cell death in the majority of cell types. However, cell death can occur when canonical NFκB activation is delayed or blocked. Binding of TNF to TNFR1 induces the recruitment of TRADD, RIPK1, TRAF2, and cIAP1 and 2 to form complex I at the plasma membrane. cIAP1 binds to TRAF2 through both its BIR1 and its UBA domains ^8– 11^. Within complex I, cIAP1 and 2 conjugate K11-, K48-, and K63-linked ubiquitin chains to themselves and other complex I components such as RIPK1 ^12– 16^. cIAP-mediated ubiquitylation of components of complex I leads to the recruitment of the linear ubiquitin chain assembly complex (LUBAC), which in turn linearly ubiquitylates several components of the complex I, including TNFR1, TRADD, RIPK1, or NEMO ^17– 22^. Both K63-linked and linear ubiquitin chains serve as docking sites for TAB2/3/TAK1 and the IKK subunit NEMO ^23– 27^. Subsequently, TAK1 phosphorylates IKK2 and MAPK kinases ^28^, leading to the transcription of NFκB-dependent and MAPK-dependent genes that induce inflammation, proliferation, and cell survival ( Figure 1).

According to this model, ubiquitylation of RIPK1 mediated by cIAP1 and 2 and LUBAC serves as a scaffold to activate NFκB and MAPK, providing inflammatory and survival outcomes ( Figure 1). However, several reports have questioned parts of this model. For instance, in Jurkat T cells lacking RIPK1, there was no activation of NFκB in response to TNF, suggesting a requirement for RIPK1 so that TNF could activate NFκB. In contrast, in primary fibroblasts and T cells, TNF was able to activate NFκB in the absence of TRADD or RIPK1 ^24, 29– 32^. Similarly, the deletion of cIAP1/2 genes markedly delayed, but did not prevent, TNF-induced activation of NFκB in mouse embryonic fibroblasts (MEFs) ^11, 33^. Observations such as these have led to a proposal that TNF induces two waves of IKK activation occurring a few minutes apart ^34^. The first is dependent on RIPK1 ubiquitylation and the second on LUBAC recruitment, which allows further recruitment of IKKs ^34^. It is therefore plausible that the first early wave has at times been missed and this could explain why in some cell types RIPK1 has been found to be dispensable for canonical NFκB activation ^34^. It also might account for why the loss of LUBAC components reduces or delays the activation of NFκB by TNF ^18– 21, 35– 39^. However, because both waves depend on TRAF2 and cIAP1, this does not explain how canonical NFκB is activated in the absence of cIAP1 and 2. Perhaps, in some cell types, in the absence of cIAP1 and 2, there are backup signaling mechanisms to ensure the transcription of survival and inflammatory genes. In other cell types, the absence of backup signaling would terminate the inflammatory response.

### TNF-induced cell death

While there remains uncertainty about whether cIAP1 and 2 are absolutely necessary for activation of the canonical NFκB pathway, there is general agreement that IAPs prevent TNF-induced cell death. Internalization of complex I leads to the recruitment of FADD, caspase-8, and RIPK3, forming a cytosolic cell death-promoting platform referred to as complex II ^40, 41^. Signaling from complex I stimulates transcription of the *CFLAR* gene encoding cFLIP, a structural homolog of caspase-8 that lacks caspase activity. Binding of cFLIP to caspase-8 limits caspase-8 activity so that a restricted number of substrates, such as RIPK1, are cleaved whereas others, such as pro-caspase-3 or Bid, are not ^42– 45^ ( Figure 1).

Cleavage of RIPK1 is believed to allow the dissociation of complex II and also prevents RIPK1 from oligomerizing with RIPK3 (another substrate of the cFLIP/caspase-8 heterodimer). Accordingly, if caspase-8 activity is compromised, uncleaved RIPK1 and 3 oligomerize to form a complex called the necrosome, in which RIPK3 is auto-phosphorylated and in turn phosphorylates MLKL, which causes a form of cell death known as necroptosis ^41, 46, 47^. Consistent with the role for cIAP1 and 2 as ubiquitin ligases for RIPK1, the absence of, a decrease in, or mutation of the BIR or UBA domains of cIAP1 and 2 allows RIPK1 to remain or become deubiquitylated, so that complex I more rapidly transitions into complex II, promoting cell death ^11, 15, 48^. This would have an immediate effect in addition to the slower effect of reduced activation of NFκB leading to less production of cFLIP ( Figure 1).

In some circumstances, the absence of just XIAP can also allow TNF to induce cell death, but less is known about how this occurs, compared with induction of cell death in the absence of cIAP1 and 2 ^49, 50^. It has been speculated that XIAP blocks RIPK1 ubiquitylation within complex II in a RIPK3-dependent manner ^50^. Consistent with this, ubiquitylated RIPK1 species are present in complex II or the necrosome when all IAPs are inhibited ^51^. Perhaps cIAP1 and 2 ubiquitylate RIPK1 in complex I, thus limiting RIPK1’s entry into complex II, whereas XIAP limits RIPK1’s ubiquitylation within complex II to block its activation.

It is important to note that, in some cells, inhibition of cIAP1 and 2 can allow spontaneous formation of a RIPK1/FADD/caspase-8/FLIP complex called the “ripoptosome” independently of the addition of TNF or other death ligands such as TRAIL and FasL ^52, 53^. Formation and activation of this complex are further enhanced in the absence of XIAP ^52– 54^ ( Figure 1). Whereas the role of cIAP1 and 2 in limiting ripoptosome formation is likely to be due to decreased RIPK1 ubiquitylation, the exact role of XIAP in inhibiting ripoptosome formation is not known.

## IAPs and microbial sensors

### Toll-like receptors and inflammasomes

Toll-like receptors (TLRs) recognize pathogen-associated and damage-associated molecular patterns known as PAMPs and DAMPs, respectively. TLRs transduce signals through the adaptor proteins MyD88 and TRIF ^55^. When PAMPs and DAMPs bind TLRs, those dependent on MyD88 recruit TRAF6, and the TRIF-dependent TLRs recruit both TRAF6 and TRAF3. TRAF6 activates NFκB and MAPK, whereas TRAF3 is believed to decrease activity of the MAPK signaling pathway and mediate IRF3-dependent production of type 1 interferon (IFN) ^55^. It has been proposed that, in the MyD88 complex, TRAF6 K63 ubiquitylates cIAP1 and 2 ^56, 57^. By K48 ubiquitylating TRAF3, cIAP1/2 cause its degradation and limit MAPK-dependent production of cytokines and chemokines without affecting the production of type I IFN ^56^ ( Figure 1). Another study suggested that cIAP1 and 2 function in concert with TRAF2/3 to mediate the degradation of c-Rel and IRF5, limiting the production of pro-inflammatory cytokines ^58^.

Although there are only a few reports on the role of IAPs in regulating TLR-induced production of cytokines, there is a substantial body of work showing that IAPs block cell death induced by TLRs. IAPs prevent TLR-dependent ripoptosome formation and a TLR-dependent, inflammasome-induced form of cell death termed “pyroptosis”. TRIF is the adaptor that links TLRs to the ripoptosome, mediated by its RIP homotypic interaction motif (RHIM) domain that binds to the RHIM domains of RIPK1/3 ^59^. Upon TLR stimulation, formation and activation of the ripoptosome are limited by IAPs ^52, 60^. Accordingly, inhibition of IAPs by Smac-mimetics sensitizes cells to TLR-induced apoptosis and necroptosis ^54, 59– 63^ ( Figure 1).

IAPs also play key roles in limiting pyroptosis, but there are conflicting views on how they do so. The nucleotide-binding oligomerization domain (NOD)-like receptor (NLR) family constitutes some of the sensors that trigger the formation of inflammasomes when they are bound by PAMPs or DAMPs in the cytoplasm. Their oligomerization results in (a) caspase-1 activation leading to pyroptosis to clear stressed cells and pathogens and (b) cleavage of interleukin-1 beta (IL-1β) to alert the immune system. Whereas Labbé *et al*. ^64^ found that cIAP1 and 2 are obligatory for IL-1β processing, other groups failed to find a role for cIAP1 and 2 in IL-1β maturation but instead showed that XIAP together with cIAP1 and 2 acted in the opposite way by preventing the cleavage of IL-1β ^50, 54, 63– 66^. Labbé *et al*. showed that in macrophages cIAP1 and 2 K63 ubiquitylate caspase-1 to enhance NLRP3 and NLRC4 inflammasome activity ^64^. In contrast, other groups found that all three IAPs limit caspase-8-dependent activation of IL-1β processing ^50, 54, 63, 65, 66^.

In macrophages, dendritic cells, and neutrophils bearing XIAP-null or -RING mutations, TLR ligation was sufficient to drive IL-1β maturation and NLRP3 activation ^50, 54, 63, 65, 66^. TLR stimulation promoted the expression of both TNF and TNFR2 ^63^. Binding of TNF to TNFR2 caused cIAP degradation ^63, 67^. Thus, in the absence of XIAP, TNFR2-induced cIAP degradation allows the formation of TNFR1-dependent complex II as well as the TRIF-dependent ripoptosome ^50, 54, 63, 65, 66^. In these complexes, activated caspase-8 leads to IL-1β processing by both NLRP3-dependent and -independent mechanisms ^50, 54, 63, 65, 66^ ( Figure 1). Accordingly, combined depletion of XIAP and cIAP1 and 2 profoundly enhanced IL-1β cleavage ^50, 54, 63, 65, 66^. Importantly, RIPK3 was also required for IL-1β processing when IAPs were inhibited ^50, 54, 65, 66^. Upon IAP inhibition, RIPK3 seems to enhance caspase-8 activity and the consequent processing of IL-1β ^63, 65^.

The differences in these observations could be due to the different genetic backgrounds of the knockout mice. Labbé *et al*. used cIAP1/2 knockout mice generated in a 129/sv background. 129/sv strains also carry a passenger mutation that inactivates caspase-11. cIAP1 and caspase-11 genes are too close in the genome to be segregated by recombination, even after extensive backcrossing ^68^. Therefore, all cIAP1 (and potentially cIAP2) knockout mice generated in a 129/sv background are likely to be mutant for caspase-11 ^68^. Because caspase-11 can cleave IL-1β in the NLRP3 non-canonical inflammasome, the effect seen in 129/Sv cIAP1 (and potentially cIAP2) knockouts might be due to non-functional caspase-11 ^69^.

Although the main activator of IL-1β is caspase-1, a role for caspase-8 in IL-1β maturation has been supported by several studies. Caspase-8 can mediate cleavage and secretion of IL-1β downstream of TLR and Fas signaling pathways, in the context of bacterial or fungal infections, during endoplasmic reticulum stress, or upon chemotherapeutic drugs ^70– 77^. None of these reports showed that IAPs had to be absent for caspase-8 to process IL-1β. However, it is worth keeping in mind that TLR signaling drives the expression of TNFR2, which in turn induces the degradation of cIAP1 and 2 ^63^. Therefore, it is plausible that bacteria or fungi that trigger TLRs reduce cIAP1/2 levels, leading to activation of complex II and the ripoptosome. In addition, some pathogens, such as *Shigella*, can both directly and indirectly inhibit cIAP1/2 functions, leading to activation of the inflammasome ^78, 79^. Similarly, it has been shown that chemotherapeutic drugs, such as etoposide, decrease IAP levels and trigger ripoptosome formation ^53^. A role for RIPK3 in promoting IL-1β processing has also been reported by others ^73, 80^. The exact molecular mechanism by which RIPK3 regulates IL-1β processing is still under investigation, but there is evidence that, in the absence of IAPs, RIPK3 favors ubiquitylation of RIPK1 and caspase-8, which presumably facilitates their activation within complex II or the ripoptosome ^50, 51, 63, 65, 81^. All together, these studies indicate that IAPs not only restrict RIPK1’s cytotoxic function but also prevent RIPK3 from enhancing IL-1β secretion.

### NOD signaling

NOD1 and NOD2 are intracellular members of the NLR family that recognize bacterial peptidoglycan derivatives. Their ligation leads to NFκB- and MAPK-dependent production of inflammatory mediators. IAPs regulate the NOD1/2 signaling pathway, and XIAP is the key player. Just as TNFR1 triggers cIAP1/2-mediated ubiquitylation of RIPK1 in complex I, NOD2 stimulation induces XIAP-mediated RIPK2 polyubiquitylation, which serves as a platform to recruit TAK1 and the IKKs ^82– 84^. XIAP interacts via its BIR2 with the kinase domain of RIPK2 to ubiquitylate RIPK2, presumably on lysines 209, 410, and 538 ^84– 88^. RIPK2 ubiquitylation by XIAP recruits LUBAC, which in turn linearly ubiquitylates RIPK2, increasing the recruitment of IKK subunits ^89^ ( Figure 1). Accordingly, deficiency of XIAP in mice completely abrogates NOD1/2 signaling and reduces responses to *Listeria* and *Chlamydophila pneumoniae* infections ^49, 89, 90^. Importantly, several studies have demonstrated that the ortholog of XIAP in *Drosophila*, DIAP2, is essential to resist Gram-negative bacterial infection ^91– 95^. These studies in flies demonstrated an evolutionarily conserved function of XIAP in regulating innate immunity.

It has been reported that, in addition to XIAP, cIAP1 and 2 can mediate the ubiquitylation of RIPK2; however, the exact role of this ubiquitylation in NOD2 signaling is still under debate ^86, 89, 96– 98^. Although there is general agreement that cIAP1 regulates NOD1/2 signaling, the mechanisms proposed differ. Bertrand *et al*. proposed that cIAP1/2-mediated ubiquitylation of RIPK2 favors activation of NFκB and MAPK induced by NOD2 ^96^. In contrast, although several studies showed that loss of cIAP1 and 2 affects RIPK2 ubiquitylation ^86, 89, 96^, two groups did not find evidence that cIAP1 and 2 directly regulate NOD2-induced NFκB and MAPK activation ^86, 98^. Instead, it has been proposed that cIAP1 increases NOD2-induced cytokine production through a TNFR1 signaling pathway ^98^ ( Figure 1). Consistent with this, TNFR1 knockout mice have a blunted response to NOD2 stimulation ^98^. As with the discordant views of how cIAP1 and 2 regulate the inflammasome, these discrepancies might be due to the use of cIAP knockouts generated in different genetic backgrounds (129/sv versus C57BL/6J) and the use of different methods to stimulate NOD signaling (DOTAP versus IFNγ priming) ^96, 98^.

Conversely, there is no doubt that XIAP plays an essential role in NOD signaling. Its importance is reflected by the existence of *XIAP* mutations contributing to human diseases in which defects in NOD signaling play a role in the pathogenesis ^86, 99– 101^. XIAP deficiency in humans causes a rare immunodeficiency syndrome characterized by high susceptibility to viruses such as Epstein–Barr virus (EBV), cytomegalovirus (CMV), or herpesvirus 6 ^102^. This syndrome is frequently referred to as X-linked lymphoproliferative disease 2 (XLP2) because the first reported XLP2 patients showed a susceptibility to EBV infections like that in XPL1 patients ^103^. However, this classification is currently under debate because so far no reported XIAP-deficient/XLP2 patient has developed lymphomas ^104– 107^. XIAP-deficient patients are affected with a range of immunological defects that can occur independently of each other. These include hemophagocytic lymphohistiocytosis, recurrent splenomegaly, and inflammatory bowel disease (IBD) resembling Crohn’s disease ^102^. Given that *NOD2* mutations are the strongest genetic factor associated with Crohn’s disease ^108, 109^, the pathological mechanism underlying IBD in XIAP deficiency is likely to be due to impaired NOD signaling. Accordingly, like those from NOD2-associated Crohn’s patients, cells from XIAP-deficient patients have reduced responses to NOD2 activation ^86, 99– 101^. On the other hand, it has been shown that NOD signaling can sense viral products ^110, 111^. Thus, the impaired response to NOD signaling in XIAP-deficient patients might contribute to their susceptibility to viral infections.

Nevertheless, defects in NOD signaling do not account for all of the signs and symptoms seen in XIAP deficiency. The role for XIAP in regulating apoptosis and the inflammasome also appears in other clinical manifestations. For instance, adaptive and innate-like T lymphocytes from XIAP patients are more sensitive to cell death induced by death receptors *in vitro* ^103, 104, 106^. This propensity to apoptosis might compromise immune responses during viral infections. In addition, it is important to note that, although there is no direct proof of a role in aberrant inflammasome activation, some XIAP patients had high levels of IL-18 in their bloodstream ^112^. Like IL-1β, IL-18 is cleaved and released upon inflammasome activation. Given that loss of XIAP in mice can activate the inflammasome, it is plausible that loss of XIAP function in these patients drives the secretion of IL-18 and the consequent associated inflammatory phenotypes.

## IAPs and tissue homeostasis

### Gene deletion

Different genetic knockouts and mutants of murine genes for XIAP, cIAP1, and cIAP2 have revealed that they work in overlapping and partially redundant ways to ensure proper embryonic development and tissue homeostasis. Mice lacking cIAP1 or 2 or XIAP are viable with no overt phenotype ^48, 113, 114^. However, unlike the co-deletion of *Xiap/Birc4* and *Ciap2/Birc3*, which leads to viable mice, co-deletion of *Ciap1/Birc2* and *Ciap2/Birc3*, or co-deletion of *Ciap1/Birc2* and *Xiap/Birc4*, results in early embryonic lethality on a pure C57BL/6 background ^48^. This suggests that cIAP1 alone is enough to achieve all essential IAP functions and also that XIAP can co-operate with cIAP2 to accomplish cIAP1’s functions. The lethality of mice lacking both XIAP and cIAP1 was nevertheless surprising. Because of the close linkage of cIAP1 and 2 genes (~15 kb), it has been assumed that they were the result of gene duplication and therefore might have redundant functions. Consistent with this idea, mice lacking XIAP and cIAP1 in a 129/Sv background are viable ^115^. These opposite results might be due to the different genetic background of the mice. Thus, it is plausible that passenger mutations such as the mutation on *caspase-11* account for 129/Sv *Xiap ^−/−^Ciap1 ^−/−^* viability. Conversely, Heard *et al*. found that the level of cIAP2 in the C57BL/6J *Xiap ^−/−^Ciap1 ^−/−^* MEFs was greatly reduced, which could explain why C57BL/6J *Xiap ^−/−^Ciap1 ^−/−^* mice were not viable ^115^. The reason for the difference in cIAP2 levels between these two sets of *Xiap ^−/−^Ciap1 ^−/−^* MEFs is still unclear. The use of CRISP/Cas9 technology might help to determine whether cIAP2 can compensate cIAP1 when XIAP is absent ^115, 116^.

The lethality of *Ciap1 ^−/−^Ciap2 ^−/−^* and *Xiap ^−/−^Ciap1 ^−/−^* mice (on a pure C57BL/6J background) occurs at E10.5 and is caused by hemorrhages and cardiovascular failure ^48^. Similar lethal defects arose in *Fadd ^−/−^*, *Cflar ^−/−^*, *Casp8 ^−/−^*, *Hoip ^−/−^*, and *Hoil ^−/−^* mutant mice ^35, 38, 48, 117– 119^. Importantly, loss of *Casp8* or *Hoip* just in the endothelia phenocopied the E10 lethality, demonstrating that the cardiac defect and hemorrhages were due to an endothelial defect ^35, 120^. Because IAPs, FADD, FLIP, caspase-8, and HOIP/HOIL all participate in the regulation of cell death induced by TNFR1, a common mechanism dependent on TNFR1 might account for the lethality in all of these knockouts. Accordingly, loss of *Tnfr1* delayed *Fadd ^−/−^*, *Casp8 ^−/−^*, *Hoil ^−/−^*, *Hoip ^−/−^*, and *Ciap1 ^−/−^Ciap2 ^−/−^* lethality, suggesting that the TNFR1-mediated endothelial cell death is responsible for some defects during embryogenesis in all of these knockouts ^35, 38, 48, 120, 121^. It is important to note that deletion of cIAP1 in zebrafish leads to endothelial cell death, implying an evolutionary conservation of the functions of IAPs ^122^.

Since cIAP1 and 2 ubiquitylate RIPK1, it seemed likely that lethality in IAP knockouts was due to aberrant TNFR1-mediated RIPK1 activation. Surprisingly, *Ripk1* loss rescued *Ciap1 ^−/−^Ciap2 ^−/−^* mice only to E12. Several subsequent reports helped explain why *Ripk1* loss did not prevent the defects seen in cIAP1/2 double mutants. Within complex II, cleavage of RIPK1 by the caspase-8/cFLIP heterodimers prevents RIPK1 from triggering full processing and activation of caspase-8 ^44^. Furthermore, binding of RIPK1 to RIPK3 via their RHIM domains prevents RIPK3 activation by other RHIM domain-containing proteins such TRIF or DAI ^123, 124^. This implies that the *Ciap1 ^−/−^Ciap2 ^−/−^Ripk1 ^−/−^* mice might die because of overwhelming caspase-8-dependent apoptosis and RIPK3-dependent necroptosis. Consistent with this idea, mutants lacking the other RIPK1 E3 ligases, *Hoil* and *Hoip*, which die at E10 from a cardiac defect similar to that in the *Ciap1 ^−/−^Ciap2 ^−/−^* mice, are rescued by co-deletion of genes for RIPK1, RIPK3, and caspase-8 ^38^. Whether the combined absence of RIPK1, RIPK3, and caspase-8 would rescue *Ciap1 ^−/−^Ciap2 ^−/−^* double mutants needs to be determined but would show whether cIAP1 and 2 act at the same level as HOIL/HOIP.

Although XIAP deficiency causes primary immunodeficiencies in humans, *Xiap ^−/−^* mice are healthy ^113^. This difference might be due to the fact that environmental factors such as pathogens also play a role in the pathogenesis of human diseases. Accordingly, unchallenged *Xiap ^−/−^* mice housed in clean facilities with controlled environments had no phenotype, but when they were challenged with pathogens they developed syndromes resembling those in XIAP-deficient patients, such as splenomegaly and increased cytokine production ^49, 50, 90, 125^. The role for cIAP1 and 2 in the gut was recently investigated. The authors showed that levels of IAPs were particularly low in enterocytes, consistent with their susceptibility to TNF-induced cell death ^126^. Whereas responses to TNF were similar in intestinal epithelial cells from wild-type, *Xiap ^−/−^*, and *Ciap2 ^−/−^* mice, those from *Ciap1 ^−/−^* mice died much more readily, highlighting a critical role for cIAP1 in intestinal homeostasis during infection ^126^. This implies that although an association of cIAP1 mutations with IBD has not been reported, low levels of cIAP1 might contribute to TNF-mediated enteropathies.

### Insights from tissue-specific knockouts

Tissue-specific IAP knockout mice have provided insights into how IAPs regulate inflammation in particular tissues. For instance, combined deletion of genes for cIAP1 and 2 in the myeloid lineage is sufficient to cause a mild inflammatory phenotype characterized by splenomegaly with disrupted splenic architecture and arthritis ^63, 127^. Although the loss of both XIAP and cIAP1 in mice did not cause any overt phenotype, the combined loss of both cIAP1/2 and XIAP severely worsened the pathology seen in myeloid-specific *Ciap1* ^−/−^ *Ciap2* ^−/−^ mice ^63, 127^. The sterile inflammation was associated with abnormally high levels of cytokines and chemokines in the bloodstream ^63, 127^. *In vitro* studies in macrophages revealed that the absence of IAPs leads to the spontaneous production of cytokines, including TNF ^127^. Furthermore, this cytokine production depended on the presence of both RIPK1 and 3, which subsequently activated apoptosis and necroptosis ^63, 65, 127^. In addition, lipopolysaccharide challenge of *Ciap1* ^−/−^ *Ciap2* ^−/−^ macrophages triggered IL-1β secretion and pyroptosis in a RIPK3-dependent manner ^63, 65^. All together, these findings demonstrated that all three IAPs repress RIPK1/3-mediated cytokine production and cell death. Many other studies have proposed that RIPK1 and 3 control cytokine production in different inflammatory settings, yet the exact molecular mechanisms remain enigmatic ^127– 131^.

Strikingly, the deletion of both cIAP1 and 2 in the epidermis induced a lethal skin inflammation that occurred in the first week after birth ^132^. Although the loss of cIAP1 in the skin combined with the loss of XIAP did not induce a lethality, these mice developed skin inflammation in adulthood ^132^. Similarly, injection of a pan Smac-mimetic into the skin of adult mice led to the development of inflammatory skin lesions ^132^. These findings highlight a vital role for these proteins in skin development and homeostasis, in which cIAP1 plays a major role. Interestingly, the early lethality of mice lacking both cIAP1 and 2 in the skin phenocopied the effects observed in skin-specific knockout of *Fadd*, *Casp8*, *Hoil*, and *Hoip* ^39, 133, 134^. All of these skin knockout mice developed epidermal hyperplasia accompanied by the death of keratinocytes and high levels of cytokines in the skin ^132– 134^. Remarkably, the loss of one allele of *Ripk1* delayed the death of the *Ciap1 ^−/−^Ciap2 ^−/−^* epidermal knockouts to weaning and completely inhibited skin inflammation caused by Smac-mimetic injection ^132^. Importantly, like the loss of cFLIP in the skin of adult mice, depletion of all IAPs with Smac-mimetic in adult mice led to skin lesions resembling a human inflammatory skin disease called toxic epidermal necrolysis ^132, 135^. On one hand, an inactivating mutation on the gene encoding the LUBAC component SHARPIN caused a form of dermatitis with features seen in psoriasis and eczema ^136^. Just as the deletion of one allele of *Ripk1* greatly reduced the severity of the lesions in skin lacking cIAP1 and 2, it also significantly delayed the *Sharpin* mutant skin phenotype ^132^. Importantly, crossing to mice bearing a mutation that inactivated RIPK1’s kinase activity provided a complete rescue of the *Sharpin* mutant phenotype ^137^. Collectively, these findings provide the rationale to test RIPK1 inhibitors in inflammatory skin diseases. In this line, GlaxoSmithKline (Brentford, UK) has an ongoing clinical trial testing RIPK1 inhibitors for the treatment of psoriasis (ClinicalTrials.gov Identifier: NCT02776033).

The deletion of genes for cIAP1 and 2 in B cells did not induce lymphocyte cell death but instead provided a survival advantage ^138^. The accumulation of B cells *in vivo* was thought to be caused by the activation of the non-canonical NFκB pathway. This pathway is activated by a subset of TNFR members and relies on the stability of the NFκB-inducing kinase (NIK). In cells not exposed to cytokine, the degradation of NIK is triggered by its ubiquitylation by a complex of TRAF2 and 3 and cIAP1 and 2 ^56, 139– 142^. Consistent with this, the mutation or deletion of genes for TRAF2 or 3 or cIAP2 has increased levels of NIK, leading to spontaneous activation of non-canonical NFκB and abnormal accumulation of B cells ^138, 143, 144^.

## Therapeutic interventions targeting IAPs

### Targeting IAPs to treat inflammatory and infectious diseases

Activating mutations in NOD2 have been associated with early onset sarcoidosis and Blau syndrome as well as early onset IBD ^145, 146^. Different strategies have been proposed to target NOD2 signaling to treat these diseases. Several groups showed that kinase inhibitors targeting RIPK2 can inhibit NOD signaling *in vitro* and *in vivo* and provide therapeutic responses in mouse models of multiple sclerosis and Crohn’s disease-like ileitis and also in Crohn’s and colitis patient samples ^147– 152^. The primary assumption was that these kinase inhibitors act by blocking RIPK2’s kinase activity. However, RIPK2 kinase-dead expressing cells had normal responses to NOD stimulation ^88, 152^. Thus, these inhibitors might act via an allosteric mechanism to interfere with the interaction of RIPK2 with IAPs ^148, 152^. These studies highlighted the IAP–RIPK2 interaction as a pharmacological target and prompted other researchers to generate XIAP antagonists to disrupt this interaction to block NOD signaling ^88^. In contrast to pan IAP antagonists, Smac-mimetics that preferentially target XIAP’s BIR2 domain did not induce cell death ^88^. Instead, these compounds affected XIAP–RIPK2 binding and inhibited NOD2 signaling ^88^. The promiscuity of many kinase inhibitors compared with the specificity with which the XIAP BIR2 domain regulates NOD signaling ^153^ renders XIAP antagonists particularly attractive for therapeutic intervention. However, given the role for XIAP in limiting IL-1β secretion (see the “Toll-like receptors and inflammasomes” section), it will be important to test the effect of targeting XIAP BIR2 on inflammasome activity.

Suicide of infected cells is one of the strategies that the immune system uses to limit pathogen dissemination and latent reservoirs. Recently, some studies suggested that targeting IAPs could be a therapeutic approach to kill human immunodeficiency virus (HIV)- and hepatitis B virus (HBV)-infected cells. Despite the success of anti-viral therapies, HIV persists because of long-lived, latently infected cells that hide from the immune system. The “shock and kill” treatment strategy consists of re-activating the viral replication of latent virions. The infected cells then would be killed either by the virus itself or by the patient’s immune system. It has been shown that Smac-mimetics can kill long-lived HIV infected CD4 ^+^ T cells or HIV-infected macrophages ^154, 155^. In addition, one study suggested that the activation of the non-canonical NFκB pathway because of inhibition of cIAP1 and 2 by a Smac-mimetic is able to reactivate the replication of latent viruses ^156^. Pache *et al*. showed that the non-canonical transcription factor RELB associates with the viral long terminal repeat to directly influence HIV transcription ^156^. The combination of a Smac-mimetic and latency-reversing agents can synergistically reverse latency in resting CD4 ^+^ T cells, providing the opportunity for these cells to be attacked by the immune system and/or killed by Smac-mimetics themselves ^156, 157^. All together, these findings suggest that Smac-mimetics might be used to eliminate latent HIV reservoirs, as they can simultaneously “shock” and “kill” latent infected cells.

Viral latency is also a challenge in HBV infection, as it predisposes to cirrhosis and hepatocellular carcinoma. TNF is an important cytokine promoting HBV clearance ^158, 159^. Taking advantage of the importance of TNF in HBV, Ebert *et al.* explored IAP inhibition to switch TNF-induced viral clearance to TNF-induced cell death ^158, 160^. They found that the deletion of IAP genes or treatment with Smac-mimetics induced early viral clearance ^158, 160^. IAP inhibition led to cell death of HBV-infected hepatocytes in a TNF-dependent manner with no collateral damage or liver failure. In addition, Smac-mimetics enhanced the efficacy of the standard drug used to treat HBV, entecavir ^158^. These findings led to a phase I/IIa study of the Smac-mimetic birinapant for the treatment of HBV carriers (ClinicalTrials.gov Identifier: NCT02288208). Unfortunately, this trial had to stop because of temporary cranial nerve palsies observed in the first cohort. This adverse event has also been observed in patients with cancer treated with two Smac-mimetics including birinapant, suggesting that it might not be due to HBV infection ^161, 162^ (ClinicalTrials.gov Identifier: NCT01188499).

### Targeting IAPs to treat cancer

The ability of IAPs to promote cell survival, and their elevated expression in many cancers, prompted efforts to target them to treat cancers ^163, 164^. Different approaches were adopted to inhibit IAPs. One was to develop peptidomimetics based on the region of Smac/Diablo that binds to XIAP’s BIR domains, so-called “Smac-mimetics”. Although these drugs were initially designed to target the BIR domains of XIAP because of the similarity to the BIRs of other IAPs, most Smac-mimetics also bind to cIAP1 and 2. It was originally thought that Smac-mimetics would induce cell death because their binding to IAPs would release active caspases in the cytosol. However, their mode of action is mainly via their ability to induce auto-ubiquitylation and degradation of cIAP1 and 2, which leads to activation of the non-canonical NFκB pathway with reduced signals activating canonical NFκB. Although there is no clear experimental proof, it is believed that the non-canonical NFκB pathway is responsible for autocrine TNF secretion. In the absence of cIAP1 and 2, binding of autocrine TNF to TNFR1 triggers the formation of complex II, which kills the cancer cells. To improve the efficacy of Smac-mimetics, several groups used the strategy of combining them with drugs that increase TNF secretion. Cytokines induced by some chemotherapeutic agents would be expected to act synergistically with Smac-mimetics. Similarly, the cytokine storm induced by non-pathologic oncolytic viruses increased Smac-mimetic killing ^165^. In addition, our group found that p38 inhibitors and caspase inhibitors increased TNF production in response to Smac-mimetics and consequently increased the amount of cell killing ^166, 167^. Although high levels of TNF can be a safety concern, ****these three combinations were proven to be well tolerated in mice ^165– 167^.

Mice mutant for IAPs helped determine which IAP would be best to target to find a safe therapeutic window. Targeting all three IAPs is highly inflammatory because it unleashes the inflammatory functions of RIPK1 and 3 which leads to the secretion of not only TNF but also the inflammasome-related cytokines IL-1β and IL-18 ^50, 54, 63, 65, 66^. The inflammatory phenotypes observed in the different IAP mutant mice and cells suggest that an ideal Smac-mimetic should strongly target cIAP1 and less cIAP2 or XIAP or both. This has been exemplified by the comparison of two Smac-mimetics presenting different affinities to each IAP ^168^. Indeed, the pan Smac-mimetic CompA (K _d_ <1 nM for all IAPs) was not tolerated in mice, as it caused widespread severe weight loss and skin lesions ^132, 168^. In contrast, the Smac-mimetic birinapant, which binds strongly to cIAP1 but has lower affinity for cIAP2 and XIAP, was generally well tolerated in mice and humans ^168^.

Another issue to consider is the malignancy of cell types that depend on the non-canonical NFκB pathway to survive or to proliferate or both. Like the deletion of genes for cIAP1 and 2 in B cells that caused the proliferation of B cells, the depletion of IAPs by a Smac-mimetic enhanced the survival of B lymphoma cells because of activation of the non-canonical NFκB pathway ^138, 169^. Nevertheless, this survival advantage is potentially reversed by combining the Smac-mimetic with a proteasome inhibitor ^170^.

It is unlikely that Smac-mimetics will be used on their own to treat cancer. Several combination treatments have been reported to enhance Smac-mimetic-induced apoptosis and necroptosis ^163, 164^. Necroptosis has recently emerged as a mechanism to allow killing of cells in which apoptotic pathways are blocked. Combined inhibition of IAPs and caspases triggered necroptosis in leukemia, pancreatic, colorectal, and ovarian cancer cells ^166, 171– 174^. Consistent with genetic studies, combining a Smac-mimetic with inhibition of caspase-8/cFLIP _L_ heterodimers using the clinical caspase inhibitor emricasan strongly triggered necroptosis in leukemic cells ^44, 45, 166^. Interestingly, in some samples of patients with acute lymphocytic leukemia (ALL), Smac-mimetics alone triggered necroptosis, possibly indicating that some ALL cases do not efficiently activate caspase-8 ^175^. This suggests that Smac-mimetics might provide an alternative treatment for cancers that have silenced caspase-8 ^176^.

Because it is a pro-inflammatory form of cell death, necroptosis can help trigger the immune system to attack cancers in a RIPK1-dependent manner ^177, 178^. Smac-mimetics have been shown to play a role in anti-tumor immunity in different ways. Because these drugs promote RIPK1 activation, they might promote anti-tumor immunity in part by RIPK1-dependent cytokine production and necroptosis. Accordingly, Smac-mimetics can increase the production of death ligands, IFNγ and IL-2 by immune cells, as well as sensitizing cancer cells to the produced death ligands ^179– 182^. Smac-mimetics can also enhance cytotoxic lymphocyte killing of tumor cells, decrease expression of the immune checkpoint PD1, polarize M2 macrophages into M1 macrophages, and reduce immunosuppressive T-cell functions ^181, 183– 186^. In contrast, inhibition of IAPs can increase the expression of the immune checkpoint PDL1, affect memory T cells, and polarize M1 macrophages into M2 macrophages, supporting the invasion and metastasis of tumor cells ^183, 187, 188^. All together, these finding suggest that Smac-mimetics can act as a double-edged sword in anti-tumor immunity. The challenge now is to determine which combinations are best applied to specific tumor types.

## Conclusions

The generation of IAP mutant mice has offered further insights into how these proteins coordinate innate immune responses. A large body of work has shown that, at the molecular level, IAPs regulate inflammation mainly through the ubiquitylation of RIPK1 and 2. Relatively little is known about other IAP substrates that might play important roles in inflammatory or other signaling pathways. Nevertheless, early findings suggest that IAPs regulate not only the ripoptosome, inflammasome, and apoptosome but also the autophagosome ^189, 190^. The pleiotropic roles for IAPs mean that Smac-mimetics are not simply “killer drugs” but also induce cytokine production that impacts on immune anti-tumor responses. Although several Smac-mimetics have entered clinical trials to treat cancer and infectious diseases, the identification of molecular and immune biomarkers of response to Smac-mimetics is still lacking.

## Abbreviations

ALL, acute lymphocytic leukemia; BIR, baculoviral repeat domain; cIAP1, cellular inhibitor of apoptosis 1; cIAP2, cellular inhibitor of apoptosis 2; DAMP, damage-associated molecular pattern; EBV, Epstein–Barr virus; HBV, hepatitis B virus; HIV, human immunodeficiency virus; IAP, inhibitor of apoptosis; IBD, inflammatory bowel disease; IFN, interferon; IL, interleukin; LUBAC, linear ubiquitin chain assembly complex; MAPK, mitogen-activated protein kinase; MEF, mouse embryonic fibroblast; NFκB, nuclear factor kappa B; NIK, NFκB-inducing kinase; NLR, NOD-like receptor; NOD, nucleotide-binding oligomerization domain; PAMP, pathogen-associated molecular pattern; RHIM, RIP homotypic interaction motif; RING, really interesting new gene; TLR, Toll-like receptor; TNF, tumor necrosis factor; TNFR1, tumor necrosis factor receptor 1; TRAF, tumor necrosis factor receptor-associated factor; XIAP, X-linked IAP; XLP2, X-linked lymphoproliferative disease 2
